# Four-year Experience of Videoconferencing-based Telepsychiatry Service in Patan Hospital: An Observational Study

**DOI:** 10.31729/jnma.v63i290.9215

**Published:** 2025-09-01

**Authors:** Bigya Shah, Pawan Sharma, Gaurav Bhattarai, Sulochana Joshi, Aayam Adhikari, Nidesh Sapkota, Rabi Shakya

**Affiliations:** 1 Department of Psychiatry, Patan Academy of Health Sciences, Lalitpur, Nepal; 2 Child and Adolescent Mental Health Services, North Staffordshire Combined National Health Service Trust, UK

**Keywords:** *digital health*, *mental health*, *telemedicine*, *telepsychiatry*, *videoconferencing*

## Abstract

**Introduction:**

Telepsychiatry has emerged as a valuable modality to bridge mental health gaps. Despite its huge potential in Nepal, it remains understudied. This study described the profiles and experiences of the patients using the free videoconferencing-based telepsychiatry service initiated at Patan Hospital, a public tertiary care center in Nepal, over four years.

**Methods:**

A retrospective review of clinical records and patients’ feedback about the videoconferencing-based telepsychiatry service from May 2021 to May 2025 from the existing service was conducted. Data were analyzed for descriptive statistics using Microsoft Excel 2016.

**Results:**

A total of 430 consultations were provided to 145 patients, with a mean age of 34.88±15.27 years; 79 (54.48%) were male, and 123 (84.83%) patients were from outside the Kathmandu Valley. 47 (32.41%) patients had anxiety disorders, followed by psychotic disorders in 44 (30.35%), depression in 18 (12.41%), and bipolar disorder in 14 (9.66%) patients. 69 (47.59%) patients used benzodiazepines, followed by second-generation antipsychotics (46.21%) and SSRIs (40.69%). A subset also received non-pharmacological interventions (21.38%). 49 (81.67%) patients preferred videoconferencing-based telepsychiatry service over face-to-face visits for the initial consultation, and 57 (95.0%) for follow-up visits. However, only 35% followed up.

**Conclusions:**

Our study highlights that implementing a videoconferencing service in a public hospital setting in Nepal is possible and it is preferred by psychiatry patients.

## INTRODUCTION

Telepsychiatry is the use of information and communication technology for psychiatric care, including clinical, preventive, diagnostic, and therapeutic services.^1^ Among various modalities, videoconferencing uses a synchronous mechanism.^2^ Globally, telepsychiatry has shown advantages and challenges.^3^

In Nepal, telemedicine programs began in 1988.^4^ Currently, there are helpline numbers run by the government and private institutions.^5^ Telepsychiatry surged during COVID-19 in private settings as social restrictions were imposed.^6^ Nepal has a large treatment gap in mental health.^7^ Nepal Media Survey showed that 72% Nepalese have smartphones with internet access, making telepsychiatry highly relevant.^8^ Other than a transient telepsychiatry service in B.P. Koirala Institute of Health Sciences (BPKIHS), there is no published information about the availability of service in public hospital setting during COVID-19.^9^ On May 24, 2021, Patan Hospital launched videoconferencing service to address mental health needs in a public health setting. This study aimed to describe the profiles and experiences of the patients using the free videoconferencing-based telepsychiatry service initiated at Patan Hospital over past four years.

## METHODS

This is a retrospective study of clinical records of the clinical records and feedback of the patients using videoconferencing-based telepsychiatry service from May 2021 to May 2025. It was conducted at Patan Hospital, a tertiary hospital situated in the Lalitpur district of Nepal. Ethical approval for the study was obtained from the Institutional Review Committee of Patan Academy of Health Sciences (Reference number: drs2507252054).

Details of the information regarding socio-demographic profile, medical comorbidities, family history of psychiatric disorders, substance use history, psychiatric diagnosis, treatment options, and number of follow-ups were collected from the clinical records. Information about the benefits and challenges of videoconferencing-based telepsychiatry service from the feedback forms of those patients who used the services between May 2021 and May 2025 was also collected and analyzed. The database managed in our study did not include any identifier or personal data that could be related to the identity of the patients.

The COVID-19 pandemic was a challenge to healthcare systems worldwide, including for Nepal. At the same time, there was a surge in mental health issues.^10^ Therefore, in a background of no similar existing service in Nepal, a new system was developed for videoconferencing-based telepsychiatry service after incorporating the findings from the review of literature and practices of the existing and past health services, and feedback from mental health professionals in the department.

The system of videoconferencing-based telepsychiatry service in Patan Hospital uses a minimalistic setup- a desktop with internet connection (internet speed: 125 Mbps/125 Mbps), a webcam, earphones, and a mobile with an internet connection. Any patient can directly call at +977-9828071488 during office hours for an appointment. We run the service twice a week with WhatsApp or Viber. After the online consultation, for every patient, a clinical note is filled, signed, and stamped, and a photo of the clinical note is sent back to the patient through the same social media channel. After the first consultation, every patient is requested to fill out an online feedback Google Form, which is protected in the email of the telepsychiatry unit (telepsychiatrypahs@gmail.com). The feedback form includes questions about the benefits and challenges of the videoconferencing-based telepsychiatry service from the patients. Microsoft Excel was used for statistical analysis. The descriptive statistics are presented as frequencies and percentages.

## RESULTS

During a period of four years, 430 consultations through videoconferencing-based telepsychiatry service were done at Patan Hospital. 145 patients received the videoconferencing psychiatric service. The mean age was 34.88±15.27 years, with an age range of 14 to 89 years, and 85 (58.60%) were young adults. Out of these, 79 (54.48%) were male and 66 (45.52%) were female. 123 (84.83%), came from outside the Kathmandu Valley, while only 22 (15.17%) lived within it. The patients were from all the provinces: 56 (38.62%) from Bagmati, 16 (11.03%) from Gandaki, 15 (10.34%) from Madhesh, 13 (8.97%) from Lumbini, 10 (6.90%) from Koshi, 9 (6.20%) from Sudurpaschim, 16 (11.03%) from Karnali, and 10 (6.90%) abroad. 23 (15.86%) were new cases for videoconferencing-based telepsychiatry service and 122 (84.14%) were follow-up consultations, which were cases first evaluated in opd settings or wards earlier. Each patient had an average of 2.97±3.03 consultations with a maximum of 25 consultations and minimum 1 consultation. Out of the 145 (100%) that were recommended for follow-up, only 51 (35.17%) followed up in telepsychiatry (Table 1).

**Table 1:** Sociodemographic and clinical profiles of the patients using videoconferencing-based telepsychiatry service (n=145).

Variables / Categories	n(%)
**Gender**	
Male	79(54.48)
Female	66(45.52)
Mean Age	34.88±15.27 years
Maximum Age	89 years
Minimum Age	14 years
**Age groups**	
Children and adolescents (≤19 yrs)	16(11.03)
Young adults (20-39 yrs)	85(58.60)
Middle adults (40-59 yrs)	3(22.76)
Geriatric (≥60 yrs)	11(7.59)
**Residence**	
Within Kathmandu Valley	22(15.17)
Outside Kathmandu Valley	123(84.83)
**Province**	
Koshi	10(6.90)
Madhesh	15(10.34)
Bagmati	56(38.62)
Gandaki	16(11.03)
Lumbini	13(8.97)
Kamali	16(11.03)
Sudurpaschim	9(6.20)
Abroad	10(6.90)
**Cases**	
New	23(15.86)
Follow-up	122(84.14)

Various psychiatric diagnoses were noted, (Table 2). 47 (32.41%) patients had anxiety disorders, followed by psychotic disorders 44 (30.35%), depression 18 (12.41%), and bipolar disorder 14 (9.66%). The other diagnoses were migraine 7 (4.83%), stress-related disorders 6 (4.14%), and substance use disorder, dementia, and personality disorder, each in 3 (2.07%). Past psychiatric history was present in 11 (7.57%), medical comorbidities in 19 (13.10%), family history of psychiatric disease in 8 (5.52%), and comorbid substance use in 20 (13.79%) patients. Among the pharmacological treatments, 69 (47.59%) patients used benzodiazepines, followed by second-generation antipsychotics in 67 (46.21%), SSRIs in 59 (40.69%), and beta-blockers in 27 (18.62%) patients. When the psychotropics were classified into broad groups, 107 (73.80%) patients used antidepressants (SSRI, TCA, SNRI, Mirtazapine), followed by antipsychotics (first and second generation combined) in 86 (59.31%), and benzodiazepines in 69 (47.59%) patients. Nonpharmacological treatment was provided in 31 (21.38%) of the cases (Table 2).

Of 60 responses feedbacks, 40 (66.67%) reported shorter waiting times than face-to-face visits, booking process was reported as easy by 51 (85.0%), and communication was easier by 28 (46.67%) patients. Regarding access to medication, 23 (38.34%) found it easier, and 5 (8.33%) found it harder. Preference for videoconferencing-based telepsychiatry service was highly observed: 49 (81.67%) preferred it over face-to-face visits for the initial consultation, and 57 (95.0%) for follow-up visits. Altogether, 46 (76.67%) experienced the service as convenient, and 51 (85%) would pay for the service, if necessary (Table 3).

**Table 2:** Clinical profiles of the patients using videoconferencing-based telepsychiatry service (n=145).

Clinical Variables / Categories	n (%)
Past History of Psychiatric illness	11 (7.57)
Medical comorbidities	19 (13.10)
Family history of psychiatric illness	8 (5.52)
Comorbid Substance use	20 (13.79)
**Psychiatric Diagnosis**	
Anxiety Disorder	47 (32.41)
Depression	18 (12.41)
Bipolar Disorder	14 (9.66)
Stress related disorder	6 (4.14)
Psychotic Disorder	44 (30.35)
Substance Use Disorder	3 (2.07)
Dementia	3 (2.07)
Personality Disorder	3 (2.07)
Migraine	7 (4.83)
^*^ **Pharmacological Treatment**	
Benzodiazepines	69 (47.59)
Selective serotonin reuptake inhibitor (SSRI)	59 (40.69)
Serotonin-norepinephrine reuptake inhibitor (SNRI)	12 (8.28)
Tricyclic antidepressant (TCA)	16 (11.03)
Noradrenergic and specific serotonergic antidepressant (NASSA, Mirtazapine)	12 (8.28)
First generation antipsychotics	8 (5.52)
Second generation antipsychotics	67 (46.21)
Mood stabilizers	19 (13.10)
Beta-blocker (Propranolol)	27 (18.62)
Anticholinergic	27 (18.62)
Anti-dementia drugs	3 (2.07)
Non-Pharmacological Intervention	31 (21.38)
Yes	51 (85.00)
No	9 (15.00)

* More than one response

**Table 3:** Patients’ experiences of using videoconferencing-based telepsychiatry service when compared with face-to-face (F2F) consultations (n=60)

Variable / Category	n (%)
**Waiting time**	
Short	40 (66.67)
Similar	8 (13.33)
Long	12 (20.00)
**Time given by psychiatrists**	
Short	8 (13.34)
Similar	26 (43.33)
Long	26 (43.33)
**Booking procedure**	
Easy	51 (85.00)
Similar	4 (6.67)
Difficult	5 (8.33)
**Clarity of conversation**	
Less clear	7 (11.66)
Similar	25 (41.47)
Better	28 (46.67)
**Difficulties in buying prescribed psychotropics**	
Difficult	5 (8.33)
Similar	32 (53.33)
Easier	23 (38.34)
**Preference of videoconferencing (first time)**	
Yes	49 (81.67)
No	11 (18.33)
**Preference of videoconferencing (follow-up)**	
Yes	57 (95.00)
No	3 (5.00)
**Overall convenience of using the service**	
Difficult	1 (1.67)
Similar	13 (21.67)
Easy	46 (76.67)
**Willingness for paid online service**	

## DISCUSSION

This study describes the profiles and experiences of the patients using the free videoconferencing-based telepsychiatry service, initiated at Patan Hospital in May 2021, over the past four years. It is the only service currently running to date since the first wave of the COVID-19 pandemic in a public hospital setting in Nepal.

The mean age was 34 years, and the majority were adults, similar to a study on telepsychiatry service from a tertiary hospital in Eastern Nepal.^9^ This might be because the younger population has more digital technology literacy than the older population in Nepal.^11^ Substantial barriers to telepsychiatry for the older population, like difficulty with technology and video platforms, hearing and language difficulties, and a lack of desire to see providers virtually exist.^12^ Similarly, children and adolescents lack privacy from caregivers as they need support for using the service.^13^

There was a slight male preponderance (54.48%), like in a study from Nepal^9^ and Bangladesh.^14^ Such gender gaps in telepsychiatry usage reveal gender gaps in health awareness and health literacy. ^14^ Further, women lag behind men in access to media devices and digital skills in Nepal.^11^ However, our finding is in contrast with another study from Bangladesh.^15^ The difference could be due to the different telepsychiatry model used in the latter study.

The location of the hospital and high adoption of digital platforms in Bagmati province, compared to other provinces, could be one of the reasons why we may have served more patients from Bagmati province.^16^ Madhesh and Karnali Provinces, with lower literacy rates and limited access to electricity and local media outlets, show lower digital platform use.^16^ However, in our study, apart from Bagmati and Gandaki provinces, we have served patients from Karnali and Madhesh provinces the most. 84.48% of the patients were outside the Kathmandu Valley, and 6.90% of our patients were also from abroad. This shows wide patient coverage of the hospital in general, also reflected in our videoconferencing-based telepsychiatry service.

Anxiety disorders were the most common diagnosis, followed by psychotic disorder, depression, and BPAD. Our findings are in congruence with studies from Bangladesh, Australia, and India.^15,17,18^ COVID-19 mental disorders collaborators from 204 countries reported that the prevalence and burden of depression and anxiety have significantly increased during the pandemic.^19^ Also, they are common mental disorders that are commonly diagnosed at health facilities. Further, 30% of patients had a diagnosis of a psychotic disorder in our study, and the finding is striking. A possible explanation could be that patients with psychotic symptoms and disorders experience lesser stigma, convenience, and ease in communicating with psychiatrists using videoconferencing-based telepsychiatry service.^20^

The most prescribed psychotropic drug was Benzodiazepines 69 (47.59%), which was similar to other studies.^9,15^ Similarly, antidepressants (SSRI, TCA, SNRI, Mirtazapine) (68.28%) were most commonly prescribed, followed by antipsychotics (51.73%) and benzodiazepines (47.59%). These findings of our study are similar to the study conducted in Nepal during the COVID-19 pandemic, but differ from the study conducted in Bangladesh, with higher use of antipsychotics in our study (51.73% vs 22.5%). ^9,15^ The difference may be due to the higher volume ofpatients with a psychotic disorder in our study (30.35% vs 14.4%). 119 (82.06%) of the patients received combination psychotropics, which was similar to a retrospective study done in Bangladesh.^15^ Non-pharmacological treatment was provided in 21.38% of the cases in the form of supportive psychotherapy sessions, motivation enhancement therapy, breathing exercises, psychoeducation about sleep hygiene, triggers of migraine, reducing screen time, and lifestyle modifications. Our findings are similar to the study by Shakya et al., in which psychoeducation sessions were reported as 22.12%.^9^ Future studies should explore the feasibility of psychotherapy in the global south, with a focus on the perspectives of therapists and patients on various psychiatric disorders.

Most of the patients who sought treatment through the videoconferencing-based telepsychiatry service were our follow-up cases. Only 15.86% had taken consultation for the first time to the hospital through the telepsychiatry service. This imparts the necessity to be aware of the public about the availability of the service in our hospital.

In general, the service users of the videoconferencing-based telepsychiatry service were positive about the experience, similar to the study by Natsukari et al.(2025).^3^ In our study, two-thirds of the patients stated that the waiting times were less than those at face-to-face (F2F) clinics. This can be explained by the fact that the majority of the patients resided outside of Kathmandu valley and their total time spent on travel lessened significantly. This finding is also in keeping with another study.^21^ A vast majority of patients (86%) reported that the time allocated for them by clinicians during the videoconferencing-based telepsychiatry consultation was either similar to or more than that of F2F consultations. It may be because we served no patients at all to a maximum of six per day, whereas about 130150 patients visit the psychiatry OPD in Patan Hospital.

Most of the service users (85%) also stated that the booking procedures were easier than the F2F consultation. This is possibly because of the unavailability of online booking or booking through phone for the psychiatry OPD. We could also see that the clarity of the conversation online was noted to be either the same as or better than the F2F consultation by most of the users (88%). This demonstrates the availability of the internet even in more rural areas of the country, with an average mobile internet speed of 13.49 Mbps.^22^ Further, psychiatry OPDs can be crowded and noisy. Only a small number (8%) reported more difficulty in acquiring the prescription than following a F2F consultation, which could possibly be due to remote location and unavailability of certain medications at their local pharmacy. However, it needs further exploration.

Only around 18 % of the service users stated they would prefer the F2F consultation for the first appointment compared to the videoconferencing-based telepsychiatry service. The preference for F2F consultations fell further to only 5% for follow-up consultations. This finding is similar with another study.^23^ However, in our study, only 35% had follow-up consultations in contrast to the advice for all. Therefore, future studies are required to study the poor retention rate in videoconferencing-based telepsychiatry services structurally.

More than 3/4 th of the service users shared that they found the videoconferencing-based telepsychiatry consultations more convenient. This highlights that for a certain group of patients, videoconferencing-based telepsychiatry service remains a convenient method of consultation. The findings are similar to those of other studies.^21,24^ However, we need more research to ascertain the generalizability of the findings, as some studies have shown a preference for F2F consultations to telemedicine in certain service user populations.^23,25^

Our service was free of cost. This could have influenced the response of the participants. However, 85% of the service users were willing to pay for the service. However, a qualitative study done in Pakistan found that the patients felt the need for the teleconsultation fee to be reduced.^6^ Some studies report that clinicians report difficulty using the telepsychiatry service despite reporting positive clinicians’ satisfaction.^13,27,28^ Nepal Medical Council guidelines also state that the payment fee structure should take into account various patients’ and clinicians’ factors.^29^ So, it is important to consider all these patients’ and clinicians’ factors while designing the telepsychiatry services fee structure.

Our study has several limitations. It is a retrospective survey conducted in a single center in a tertiary public hospital and initiated during the COVID-19 pandemic, and was free of cost. This study may not be generalizable to other times, rural, and private settings. Many were follow-up cases of F2F consultations, less than half ofthe participants provided feedback, no standardized tool was used to measure the effectiveness, and a few follow-ups limit our ability to conclude on the effectiveness of videoconferencing services. However, being the first of its kind of service currently being run in a public setting since the first wave of the COVID-19 pandemic, our videoconferencing-based telepsychiatry services can serve as a model for scaling up telepsychiatry services in other parts of Nepal and in similar low-resource settings. Future studies should explore long-term clinical outcomes, cost-effectiveness, gender gaps, clinicians’ experiences, and integrating psychotherapy services and diverse psychiatric disorders with better research designs. There is a need to develop national telepsychiatry guidelines.

## CONCLUSION

Our study highlights that implementing a videoconferencing-based telepsychiatry service in a public hospital setting in Nepal is possible and holds the promise of being convenient and satisfactory for psychiatric patients. The service successfully reached a diverse population, mostly adults and follow-up cases, all over Nepal and abroad. The majority of service users preferred the service and reported positive experiences. Despite the challenges like retention rate and medicine access, it can complement the conventional face-to-face psychiatry services in Nepal.

## Data Availability

The data are available from the corresponding author upon reasonable request
